# Real time patient‐reported outcome measures in patients with cancer: Early experience within an integrated health system

**DOI:** 10.1002/cam4.5635

**Published:** 2023-01-20

**Authors:** Samantha Tam, Theresa Zatirka, Christine Neslund‐Dudas, Wan‐Ting Su, Cara E. Cannella, Jeewanjot S. Grewal, Ahmad H. Mattour, Amy Tang, Benjamin Movsas, Steven S. Chang

**Affiliations:** ^1^ Department of Otolaryngology – Head and Neck Surgery Henry Ford Hospital Detroit Michigan USA; ^2^ Division of Clinical and Quality Transformation, Transformation Consulting Henry Ford Health Detroit Michigan USA; ^3^ Department of Public Health Sciences Henry Ford Health Detroit Michigan USA; ^4^ Department of Hematology‐Oncology Henry Ford Health Detroit Michigan USA; ^5^ Department of Radiation Oncology Henry Ford Hospital Detroit Michigan USA

**Keywords:** cancer pain, healthcare disparities, oncology service, patient reported outcome measures

## Abstract

**Background:**

While patient‐reported outcome measures (PROMs) have benefit in cancer clinical trials, real‐world applications are lacking. This study describes the method of implementation of a cancer enterprise‐wide PROMs platform.

**Methods:**

After establishing a multispecialty stakeholder group within a large integrated health system, domain‐specific instruments were selected from the National Institutes of Health's Patient‐Reported Outcomes Measurement Information System (PROMIS) instruments (pain interference, fatigue, physical function, and depression) and were administered at varying frequencies throughout each patient's cancer journey. All cancer patients with an oncologic visit were eligible to complete the PROMs prior to the visit using a patient portal, or at the time of the visit using a tablet. PROMs were integrated into clinical workflow. Clinical partnerships were essential for successful implementation. Descriptive preliminary data were compared using multivariable logistic regression to determine the factors associated with method of PROMs completion.

**Results:**

From September 16, 2020 to July 23, 2021, 23 of 38 clinical units (60.5%) implemented PROMs over 2392 encounters and 1666 patients. Approximately one third of patients (*n* = 629, 37.8%) used the patient portal. Black patients (aOR 0.70; 95% CI: 0.51–0.97) and patients residing in zip codes with higher percentage of unemployment (aOR: 0.07, 95% CI: 0.01–0.41) were among the least likely to complete PROMs using the patient portal.

**Conclusions:**

Successful system‐wide implementation of PROMs among cancer patients requires engagement from multispecialty stakeholders and investment from clinical partners. Attention to the method of PROMs collection is required in order to reduce the potential for disparities, such as Black populations and those residing in areas with high levels of unemployment.

## Graphical abstract

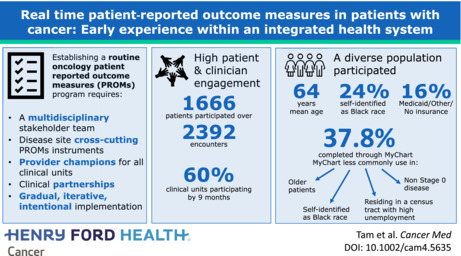

## 1 INTRODUCTION

Patients undergoing treatment for cancer can experience a wide spectrum of symptoms. While physician assessment with clinical history and examination are the traditional methods of assessing treatment toxicity and disease recurrence, there exist major gaps between physician assessment and patient experiences. Patient‐reported outcome measures (PROMs) represent a patient‐centered method to quantify the patient voice. PROMs have been demonstrated to be complementary to the physician assessment and are superior in determining disease outcome in patients with non‐small cell lung cancer.^1^, ^2^

Basch et al.^3^ utilized PROMs alone as an intervention in a randomized clinical trial of patients with metastatic solid tumors. PROMs facilitated symptom communication and management. Use of PROMs resulted in improved patient quality of life, decreased emergency room visits and hospitalization, and increased the duration of chemotherapy compared to patients undergoing usual care. In patients where PROMs were used, 1‐year overall and quality‐adjusted survival were also improved.^4^ In addition, PROMs have been demonstrated to be essential in the assessment of symptoms in multiple clinical trials.^2^, ^5^, ^6^, ^7^, ^8^ Therefore, PROMs are potentially very powerful tools that can affect a threshold change in quality of cancer care. However, implementation of PROMs is often limited to clinical trial settings. Only 2%–5% of new cancer patients enroll in clinical trials.^9^, ^10^, ^11^ Patients with increased age, in ethnic/racial minorities, and low socioeconomic status factors are less likely to enroll and represent the same population with poorer cancer outcomes.^9^, ^10^, ^12^, ^13^, ^14^ These patients also experience barriers in patient‐physician communication.^15^, ^16^ Population‐based implementation of a PROMs platform would capture patients not routinely enrolled in clinical trials and may provide a standardized symptom communication tool to improve equity in cancer care. This study aims to describe the process of initial engagement and early implementation of PROMs throughout the cancer service line at a vertically integrated metropolitan health system. To assess the implementation, we assessed the number of clinical units successfully implementing the program and describe the population of patients and method of completing PROMs to understand areas of strength and improvement for future implementation efforts.

## 2 METHODS

### 2.1 Implementation of electronic patient‐reported outcomes program

#### 2.1.1 Establishing a team of stakeholders

Henry Ford Cancer (HFC) is a tertiary care center within Henry Ford Health, a vertically integrated health system serving a diverse population throughout metropolitan Detroit and rural south‐central Michigan. Recognizing the importance of PROMs in the delivery of cancer care, the HFC Patient‐Reported Outcomes Committee was established in September 2019. The purpose of the Committee was to standardize the PROMs collection process for HFC patients while balancing survey burden and maximizing value of patient feedback during cancer treatment. Committee members represented diverse HFC specialties: surgical/radiation/hematology oncology, neuro‐oncology, supportive oncology, palliative medicine, plastic surgery, public health sciences, precision medicine, OncoStat (a specialized oncologic urgent care clinic run by advanced practice providers and registered nurses), quality, and cancer research. A critical component of this committee was the dyad leadership of physician leader, administrator, and physicians representing each specialty. To address operational components of electronic PROMs throughout the system, an HFH PROMIS Taskforce was convened in early 2019 which resulted in the launch of a PROMs module in the EHR. The taskforce included executive and operational sponsors as well as leadership and front‐line staff from the EHR and IT teams. The involvement of these partners and stakeholders was essential for sustainability and benefits realization of the project.

#### 2.1.2 Selection of PROMs instruments

Leveraging the infrastructure established by the task force, the Committee selected the National Institutes of Health's Patient‐Reported Outcomes Measurement Information System (PROMIS) as the PROMs instrument. The PROMIS computer adaptive test (CAT) version was selected due to its high level of precision and ability to measure the domain with less questions and greater accuracy than standard short forms.^7^ Four PROMIS domains were chosen: pain interference (PROMIS CAT version 1.1—Pain Interference^8^), fatigue (PROMIS CAT version 1.0—Fatigue^9^), physical function (PROMIS CAT version 2.0—Physical Function), and depression (PROMIS CAT version 1.0—Depression^10^, ^11^). Selected PROMIS measures were domain specific, rather than disease‐specific, to allow uniform application of PROMs for all cancer disease sites within the service line. These PROMs have been developed and validated to measure function and symptoms in both the general population and those with chronic conditions including cancer.^1^, ^2^, ^3^, ^4^, ^5^, ^6^ These domains were selected based on consensus from the Committee as they are impacted by cancer diagnosis and treatments for the vast majority of cancer types and stages; they can also help to facilitate clinical care and appropriate referrals. These domains were also recommended by HealthMeasures—“the dissemination and implementation hub for … PROMIS … originally funded by the National Institutes of Health” as the key domains for cancer care.^17^ Selection of cross‐cutting instruments maximized the collection of PROMs from different time points throughout the cancer care continuum and allowed future broader integration throughout noncancer care in the larger Henry Ford Health system. The Committee chose only four domains for initial implementation to be mindful of survey burden and in anticipation of cancer disease teams' desire to add disease‐specific PROMs specific to their cancer type in the future. Composite scores for each domain were reported as T‐scores. Severe scores were defined as scores >2.0 standard deviations from the mean score.^18^

#### 2.1.3 Patient identification for administration of PROMs instruments

**FIGURE 1:**
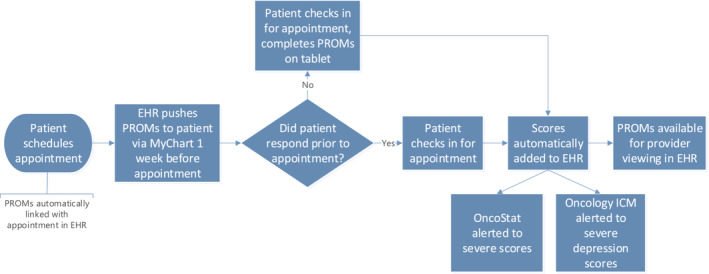
Schematic flowsheet demonstrating the process of PROMs instrument completion in an ambulatory oncology clinic. EHR, electronic health record; ICM, integrated case management; PROMs, patient‐reported outcome measures.

PROMs were automatically assigned in the electronic health record (EHR) in patients ≥18 years old with an oncologic International Classification of Diseases, 10th Revision, Clinical Modification code on their problem list seeing a surgical, radiation, medical, or supportive oncology provider. PROMs were offered prior to the visit on the MyChart (Epic Systems, Verona, WI) patient portal (Figure S1). MyChart is a patient facing interface that allows patient access to their medical records and allow for remote patient‐provider communication either through their mobile device or a web page. Patients were given a one‐week window to complete PROMs as scores were intended to be available for review prior to a visit to augment the clinical assessment. If instruments were incomplete at the time of the visit, a tablet was provided in the waiting room for completion (Figure 1, Figure S2). PROMIS measures were then instantly uploaded to the EHR for review by a clinician during the office visit. Of note, the EHR message offering PROMs reminded patients to directly seek urgent care for any urgent concerns or symptoms.

Baseline PROM scores were targeted at new oncology patient visits. PROM scores were collected once a week during active treatment (systemic or radiation therapy) for pain interference, fatigue, and physical function. Depression was collected once a month during active treatment. To reduce survey burden for patients who remain on active treatment for ≥6 months, the frequency of collection was adjusted to once every 3 months, then monthly in survivorship year 1; quarterly in survivorship year 2; every 6 months in survivorship years 3–5; and yearly thereafter (Table 1). This schedule attempts to mimic usual surveillance criteria from the National Comprehensive Cancer Network.^19^ Lookback days in the EHR were used to adjust for the desired frequency of PROMs administration. If the patient completed the PROMs instrument within the threshold, the EHR would not assign the measure to the visit. If the patient did not complete the PROMs during that time, the EHR would automatically assign the measure to the visit.

**TABLE 1:** Patient‐reported outcome measure targeted frequency according to Patient‐Reported Outcomes Measurement Information System domain

Timepoint in cancer care continuum	Domain	Frequency
New patient visit	Pain interference Fatigue Physical function Depression	Once
Active treatment	Pain interference Fatigue Physical function	Weekly
Active treatment	Depression	Monthly
Survivorship year 1	Pain interference Fatigue Physical function Depression	Monthly
Survivorship year 2	Pain interference Fatigue Physical function Depression	Quarterly
Survivorship year 3–5	Pain interference Fatigue Physical function Depression	Every 6 months
Survivorship year 6+	Pain interference Fatigue Physical function Depression	Yearly

#### 2.1.4 Implementation of PROMs instruments within clinical units

To be mindful of the impact of introducing change, change management processes were employed throughout. Thus, to facilitate clinical rollout of PROMs, clinical units were identified. Clinical units were groups of providers that worked in the same context whose clinical workflows overlapped physically. While this resulted in a more gradual implementation of the program, it ensured time for observation, adaptation, and application of lessons learned from previous clinical units to subsequent clinical units. An engaged physician champion was identified for each clinical unit. This was found to be essential to identify the specific culture of each unit. Prior to each rollout, the PROMs Committee co‐chairs met with each clinical unit for a presentation and discussion. The presentation included brief background on why PROMs should be part of standard cancer care, an introduction to PROMIS, an overview of the workflow, and, as rollouts progressed, examples of how PROMs had been additive to patient care. After a rollout date had been identified, the clinic front desk team was trained on how to use the tablets for PROM collection and provided a brief background on PROMs. On the day of each rollout, a PROMs team member was present at the clinic front desk to support team members' initial use of tablets. The PROMs team member was also available to providers to answer questions. Providers were instructed on how to access and interpret scores. Scores were used as a platform for symptom discussion and thus need for intervention was left up to the discretion of the provider. Strong support for this PROMs program by the cancer leadership is also an important factor.

#### 2.1.5 Clinical partnerships

HFC and health system partners have been essential to PROM implementation. One such partner is the HFC OncoStat Clinic—a same‐day cancer symptom management clinic staffed by oncology advance practice providers and nurses. OncoStat is alerted to all severe pain interference, fatigue, and physical function scores daily through a confidential email report pending completion of an EHR‐based notification system, which was completed after this paper's timeframe (Figure 1). This process was identical regardless of method of PROMs completion. OncoStat contacts the patient by the following business day to follow‐up on the severe PROMs score, educates the patient on OncoStat services, and offers appropriate interventions. OncoStat's utilization of PROMs scores to identify patients who may need timely follow‐up also mitigates the potential for a severe score to go unseen by an ambulatory oncology provider.

The Ambulatory Oncology Integrated Case Management team is another partner. These integrated case managers (ICM) are licensed master's social workers aligned by regional hospital and by cancer type in Detroit. The ICM team is alerted to all severe depression scores daily through a process similar to OncoStat, described above (Figure 1). The ICM contacts the patient by the following business day or meets with the patient in clinic if there is an upcoming oncology appointment the same week. The ICM provides brief supportive counseling, assesses for safety and support, and escalates to the Psycho‐Oncology team if appropriate.

### 2.2 Preliminary data and statistical analysis

PROMs were collected from September 2020 to July 2021. Following Henry Ford Health Institutional Review Board approval, data were collected retrospectively. Due to the retrospective nature of this study, requirement for informed consent was waived. To understand the patient population completing PROMs, descriptive statistics were reported. All analysis was completed on patient‐level data. Patient age at completion of PROMs, sex, race and ethnicity, zip code based socioeconomic status, insurance status, disease site, and stage were collected and compared according to the method of completion of PROMs (via MyChart vs. not MyChart) using Student's *t*‐test, Kruskal–Wallis test, and *χ* ^2^ test as appropriate. This comparison was completed to better understand both remote and in‐clinic completion workflows. Patients were defined as completing PROMs if any of the instruments were completed through either modality prior to the start of their provider visit (i.e., completion in the waiting room was also considered as completed PROMs). Multivariable logistic regression was completed to determine factors associated with successful use of MyChart to complete PROMs. Stepwise selection was used to decide on variables included in the final model using a Wald *χ* ^2^ score of *p* ≤ 0.05 for variable entry into the model and *p* ≤ 0.10 for the variable to remain in the model. Patients with missing fields in the included variables were excluded from the model. All tests were two‐tailed and a *p* < 0.05 was considered significant. All statistical analyses were completed using SAS 9.4 (SAS Institute, Cary, NC).

## 3 RESULTS

**FIGURE 2:**
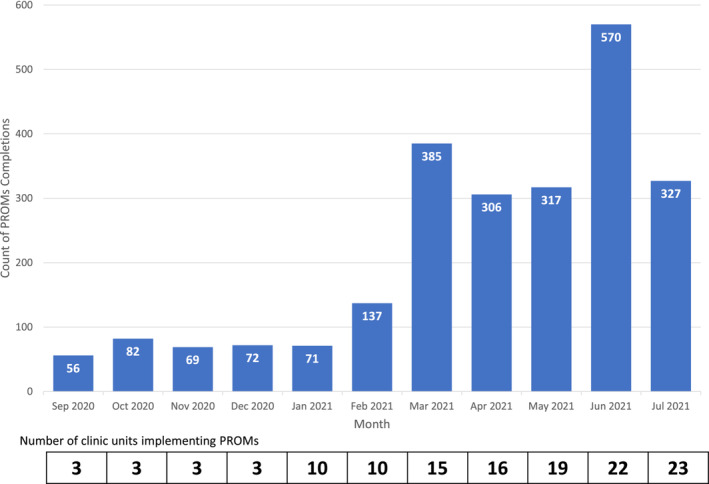
Bar graph demonstrating count of PROMs completions and clinic unit implementation by month. PROMs, patient‐reported outcome measures.

Roll‐out of the program started as a pilot in a single specialty (head and neck cancer) with seven providers. From September 16, 2020, to July 23, 2021, PROMs were implemented in 23 (60%) clinical units (60 providers), with an additional 15 units planned for the subsequent months for a total of 38 clinical units (Figure 2). These 23 clinical units were spread over three hospitals and four medical centers.

### 3.1 Patient demographics

A total of 1666 patients completed PROMs over 2392 patient encounters (Figure 2). Patient characteristics are summarized in Table 2. In total, 470 patients (28.2%) completed PROMs over more than one visit with a maximum number of completions of 12. Mean age at completion of PROMs was 64.3 years (standard deviation 12.4). Non‐Hispanic White patients comprised 67.2% of the sample (*n* = 1120), Black comprised 23.7% (*n* = 395), and Hispanic/Latino patients comprised 1.7% (*n* = 28). Most patients were insured with private insurance (*n* = 789, 47.4%). The most common cancer disease sites completing at least one PROM were breast (*n* = 490, 29.4%), head and neck (*n* = 219, 13.1%), and lung (*n* = 192, 11.3%).

**TABLE 2:** Patient characteristics of patients completing patient‐reported outcome measures from September 16, 2020, to July 26, 2021, according to use of MyChart patient portal

Characteristic	All		MyChart use (*n* = 629)		No MyChart use (*n* = 1037)		*p*‐value
	*n*	%	*n*	%	*n*	%	
Age							
Mean (SD)	64.3 (12.4)		63.4 (12.0)		64.8 (12.7)		0.0254
Sex							
Male	680	40.8	231	36.7	449	43.3	0.0081
Female	986	59.2	398	63.3	588	56.7	
Race/ethnicity							
Non‐Hispanic White	1120	67.2	468	74.4	652	62.9	<0.001
Black	395	23.7	108	17.2	287	27.7	
Hispanic	28	1.7	13	2.1	15	1.4	
Other/unknown	123	7.4	40	6.4	83	8.0	
Marital status							
Married	961	57.7	387	61.5	574	55.4	0.0134
Not married	705	42.3	242	38.5	463	44.6	
Insurance status							
Private	789	47.4	223	52.8	457	44.1	0.0017
Medicare	602	36.1	210	33.4	392	37.8	
Medicaid/other/none	275	16.5	87	13.8	188	18.1	
Charlson comorbidity score							
0	339	20.4	114	22.9	195	18.8	0.0625
1–2	416	25.0	157	25.0	259	25.0	
≥3	356	21.4	116	18.4	240	23.1	
Missing	555	33.1	212	33.7	343	33.1	
Cancer disease site							
Blood/bone	95	5.7	31	4.9	64	6.2	0.4429
Breast	490	29.4	204	32.4	286	27.6	
Central nervous system	108	6.5	40	6.4	68	6.6	
Gastrointestinal	158	9.5	57	9.1	101	9.7	
Genitourinary	194	11.6	64	10.2	130	12.5	
Gynecologic	49	2.9	20	3.2	29	2.8	
Head and neck	219	13.1	88	12.6	131	14.0	
Lung	192	1.5	69	11.0	123	11.9	
Other	161	9.7	56	8.9	105	10.1	
Stage							
Stage 0	84	5.0	45	7.2	39	3.8	0.0004
Stage I	385	23.1	164	26.1	221	21.3	
Stage II	258	15.5	88	14.0	170	16.4	
Stage III	249	15.0	102	16.2	147	14.2	
Stage IV	330	19.8	102	16.2	228	22.0	
Unknown	360	21.6	128	20.4	232	22.4	
Surgery							
Yes	945	56.7	391	62.2	554	53.4	0.0005
No	721	43.3	238	37.8	483	46.6	
Radiation							
Yes	636	38.2	247	39.3	389	37.5	0.4743
No	1030	61.8	382	60.7	648	62.5	
Systemic therapy							
Yes	704	42.3	252	40.1	452	43.6	0.1581
No	962	57.7	377	59.9	585	56.4	

Abbreviation: SD, standard deviation.

### 3.2 Method of PROMs completion

A total of 629 patients (37.8%) were able to complete at least one PROMs instrument using the MyChart patient portal (Table 2). Patients who completed PROMs instruments using MyChart were younger (63.4 years vs. 64.8 years, *p* = 0.025) and more likely to be female (40.4% vs. 34.0%, *p* = 0.0081) and married (40.3% vs. 34.3%, *p* = 0.0134) compared with patients not using MyChart. Patients were less likely to be Black (*p* < 0.001). Patients completing PROMs using MyChart were more likely to reside in zip codes with higher median household income levels (*p* < 0.0001), higher education levels (*p* = 0.0027), lower levels of poverty (*p* < 0.0001), and lower levels of unemployment (*p* < 0.0001; Table 3).

**TABLE 3:** Patient zip code based socioeconomic status characteristics according to use of MyChart patient portal

Parameter	Mean (SD)			*p*‐value
	All	Any MyChart use	No MyChart use	
Median household income (2021 United States dollars)	52,235.3 (20,613.3)	55,105.9 (20,472.6)	50,515.1 (20,515.3)	<0.0001
Percentage households below poverty level	17.4 (12.6)	15.4 (10.1)	18.7 (13.3)	<0.0001
Percentage of unemployed persons ≥16 years old	15.2 (7.7)	13.9 (6.6)	16.0 (8.2)	<0.0001
Percentage of patients with less than high school education	16.0 (8.3)	15.1 (7.4)	16.6 (8.7)	0.0027

Abbreviation: SD, standard deviation.

The final multivariable logistic regression model adjusted for age at completion of PROMs, race and ethnicity, stage, treatment with surgery, and unemployment rate (Table 4). Black patients had 0.70 less adjusted odds of completing PROMs using MyChart (95% CI 0.51–0.97) compared to non‐Hispanic White patients. Per year of increased age, patients had 0.99 fewer odds of completion of PROMs using MyChart (95% CI 0.98–1.00). Patients with Stage 0 tumors had 2.10 more odds (95% CI 1.26‐3.48) of completion using MyChart compared to those with unknown stage disease. Patients residing in zip codes with higher unemployment rates were also less likely to complete PROMs using MyChart (adjusted odds ratio 0.07, 95% CI 0.01–0.41).

**TABLE 4:** Multivariable logistic regression for completion of patients‐reported outcomes using MyChart adjusted for age at completion, race and ethnicity, stage, treatment, and unemployment rate

Characteristic	Adjusted odds ratio	95% CI		*p*‐value
		Lower limit	Upper limit	
Age	0.99	0.98	1.00	0.0246
Race				
White	Ref			0.0428
Black	0.70	0.51	0.97	
Hispanic	1.39	0.65	3.00	
Other/unknown	0.69	0.46	1.03	
Stage				
Unknown	Ref			0.0043
Stage 0	2.10	1.26	3.48	
Stage I	1.32	0.95	1.83	
Stage II	0.97	0.67	1.38	
Stage III	1.34	0.94	1.91	
Stage IV	0.87	0.63	1.2	
Treatment with surgery				
No	Ref			0.0798
Yes	1.23	0.98	1.51	
Percentage of unemployed persons ≥16 years old	0.07	0.01	0.41	0.0032

Abbreviation: Ref, reference.

## 4 DISCUSSION

The utility of PROMs has been well demonstrated in cancer for symptom management and improving cancer‐related outcomes. However, efficacy of PROMs has often been limited to clinical trial settings. Clinical trials represent time‐limited instances of PROMs implementation with funded resources to ensure success of the study. In order to translate the benefits of PROMs to the general population, a novel approach to the delivery and collection of instruments is required for integration into usual clinical workflow. Herein we have outlined essential elements required for successful system‐wide implementation of a PROMs platform across a wide breadth of cancer disease sites at HFC.

The Institute of Medicine's 1999 report on Quality in Cancer Care brought to light the chasm between the ideal and reality of delivery of cancer care. Since then, great interest in using PROMs in routine clinical care has grown. Implementation of a PROMs program in oncology has been advocated for nationally.^20^ While implementation of PROMs has been successful within disease‐specific clinics, this manuscript describes a program that is rather unique in the breadth of implementation (i.e., throughout the cancer enterprise).^6^, ^21^, ^22^ Strategies for success were engagement of multidisciplinary providers representing a wide breadth of clinical disease sites at the onset of the process, strong support from the cancer leadership, and focusing on implementation of domain, rather than disease‐site specific instruments. In addition to stakeholders, a physician champion was identified for each clinical unit. Local supervisors of clinic front desks along with Integrated Case Managers were also engaged for each unit. This allowed for understanding and process adaptation to each clinics' unique workflow and requirements. Two key contextual factors were present throughout implementation. In addition to the second and third waves of the COVID‐19 pandemic, HFC opened a new flagship cancer center in Detroit while shifting to a new model of care. With direction from HFC leadership, implementation was deliberate and gradual to account for high levels of change fatigue. This also enabled iterative cycles of implementation, review, adjustment, and application of lessons learned. Focused engagement with individual or small groups of providers, particularly early in the implementation when change fatigue was at its highest due to the aforementioned contextual factors, provided the opportunity for dialog with providers to address concerns, incorporate their feedback, and increase acceptability of the implementation.

One of the major drawbacks of PROMs implementation to clinicians is the perceived added clinical burden an additional measurement tool would bring to each patient encounter. While patients have demonstrated positive feedback with using PROMs, physicians are already overburdened, with burnout being an important issue among those practicing oncology.^23^, ^24^ PROMs may add additional complexity in the clinical encounter; while they are effective symptom reporting tools, clinical guidelines on how to treat abnormal findings are lacking. To alleviate the potential clinical burden of PROMs within HFC, essential clinical partnerships were built to leverage advanced practice providers to address concerning PROMs responses. Indeed, we uniquely view PROMs as another kind of “vital sign” that is transmitted in real‐time to our OncoStat team to address severe PROMs scores.^25^ This model has demonstrated effectiveness in clinical trial settings and may provide an additional safety net as clinical guidance on appropriate actions in response to PROMs develop.^3^, ^22^

PROMs responses in this study demonstrated completion in a racially and ethnically diverse patient population compared to some clinical trial applications of PROMs.^4^, ^26^ There are well known racial and ethnic disparity in clinical trials enrollment.^9^, ^27^, ^28^, ^29^, ^30^ As well, patient‐physician symptom communication is often lacking across racial and ethnic divides.^15^, ^16^ PROMs, as a standardized symptom reporting platform, may offer an opportunity to lessen these disparities. Qualitatively, Blacks have reported perceived value in PROMs to improve symptom communication with physicians in breast cancer, with greater reported perceived value compared to White patients.^24^ As implementation of PROMs in routine practice has demonstrated success in capturing a wide patient population, it demonstrates promise as a platform to increase equity in cancer care. Future studies investigating differences in response rate and implementation strategies to increase response in vulnerable populations have the potential to further lessen disparities.

While PROMs may act as a platform for more equitable cancer care delivery, the method of delivery of PROMs itself may widen existing divides in access to care. PROMs are delivered through a variety of methods, both on paper and digitalelectronically.^6^ Use of remote completion in this study demonstrate a digital division within our population. White, younger, female, and married patients and those with in situ disease or living in higher socioeconomic status areas were more likely to have completed PROMs prior to their visit using the patient portal. This reflects trends in electronic PROMs completion in co‐operative group clinical trial settings.^31^ Known disparities exist in patient portal uptake among those with non‐White race, increased age, and low levels of education.^32^, ^33^ The present study anchored PROMs completion with an oncologic visit, allowing in‐person completion with a tablet available at appointment check‐in as a backup to the patient portal; in‐person completion accounted for the majority of encounters. Tablets were distributed to clinic front desks based on estimated clinic volume. Clinics were provided with additional tablets when requested. By allowing for this alternate completion method, patients maintained access to PROMs even if they did not have access to technology to complete instruments remotely. Thus, while electronic completion of PROMs allows for seamless integration into the EHR, the medium on which PROMs is completed needs to be chosen strategically in order to not exacerbate existing disparities. Education interventions to increase patient portal use in vulnerable patients have demonstrated success and may allow future improvement in remote completion, which may facilitate patient‐provider communication outside of physician visits.^34^ Targeted interventions to improve PROMs have been attempted in a variety of vulnerable populations and the findings in this study can help guide future directions to improve equity in PROMs implementation within the cancer population.^35^

While integration of PROMs in usual clinical care has been successful, there continues to be limitations to this study. Importantly, further evaluation of this initial implementation is essential and currently, a mixed methods study of providers, clinical staff, and patients is ongoing to provide insight into facilitators and barriers in this initial effort. Further efforts in identifying non‐responders are required. Focused interventions engaging patients within populations at high risk of non‐response is required to ensure equitable implementation of PROMs. As PROMs was only available in English in this experience, non‐English options may also increase uptake in vulnerable populations. Translations are available for many validated PROMs and the plan is to implement as a next step. As these different strategies are implemented, further understanding on the reliability of responses in different contexts is required. While clinical partners are engaged in managing PROM responses, better understanding of the clinical implications of PROMs scores to further refine clinical pathways for patients with concerning symptoms is required. Decreasing the divide in patient portal use may also improve patient completion and clinic workflow in the future. This would also facilitate between in‐person visit assessments and may allow for further implementation of PROMs outside the oncologic context. Lastly, implementation in the last remaining clinical units and long term sustainability of this effort needs to be demonstrated.

## 5 CONCLUSIONS

While continued assessment and future refinement of this system‐wide implementation of PROMs among cancer patients is ongoing, this study demonstrates early success. Implementation was successful in a wide variety of clinical settings capturing many cancer disease sites within a diverse patient population. A digital divide continues to exist in the method of completion, especially among Black patients, older patients, and patients residing in areas of high unemployment. Further understanding of non‐response, effect of PROMs on cancer care delivery in diverse patients, physician education and action on PROMs, and long‐term sustainability still need to be explored.

## AUTHOR CONTRIBUTIONS

**Samantha Tam:** Conceptualization (lead); data curation (supporting); methodology (lead); writing – original draft (equal). **Theresa Zatirka:** Conceptualization (supporting); data curation (supporting); methodology (equal); project administration (lead); writing – original draft (equal). **Christine Neslund‐Dudas:** Conceptualization (supporting); formal analysis (supporting); writing – review and editing (equal). **Wan‐Ting Su:** Data curation (lead); writing – review and editing (equal). **Cara E. Cannella:** Data curation (supporting); writing – review and editing (equal). **Jeewanjot S. Grewal:** Conceptualization (supporting); writing – review and editing (equal). **Ahmad H. Mattour:** Conceptualization (equal); writing – review and editing (equal). **Amy Tang:** Conceptualization (equal); writing – review and editing (equal). **Benjamin Movsas:** Conceptualization (equal); writing – review and editing (equal). **Steven S. Chang:** Conceptualization (equal); methodology (lead); writing – review and editing (equal).

## CONFLICT OF INTEREST

The authors have no conflict of interest to declare .

## Supporting information


Appendix S1

Figure S1

Figure S2

## Data Availability

The data that support the findings of this study are available from the corresponding author upon reasonable request.
