# Supplementary material for: Real time patient‐reported outcome measures in patients with cancer: Early experience within an integrated health system
Source: Cancer Med. 2023 Jan 20;12(7):8860–70. doi: 10.1002/cam4.5635 (PMC10134279; doi:10.1002/cam4.5635)
Supplement: Supplementary file 1 — Appendix S1 [file CAM4-12-8860-s001.docx]

**Supplemental Materials**

**Supplemental Figure Legend:**

**Supplemental Figure 1.** Example of the patient view when completing a PROMIS instrument on the MyChart patient portal

**Supplemental Figure 2.** Example of the patient view when completing a PROMIS instrument in clinic with a clinic provided tablet.
