# Supplementary figures and images for: Real time patient‐reported outcome measures in patients with cancer: Early experience within an integrated health system
Source: Cancer Med. 2023 Jan 20;12(7):8860–70. doi: 10.1002/cam4.5635 (PMC10134279; doi:10.1002/cam4.5635)

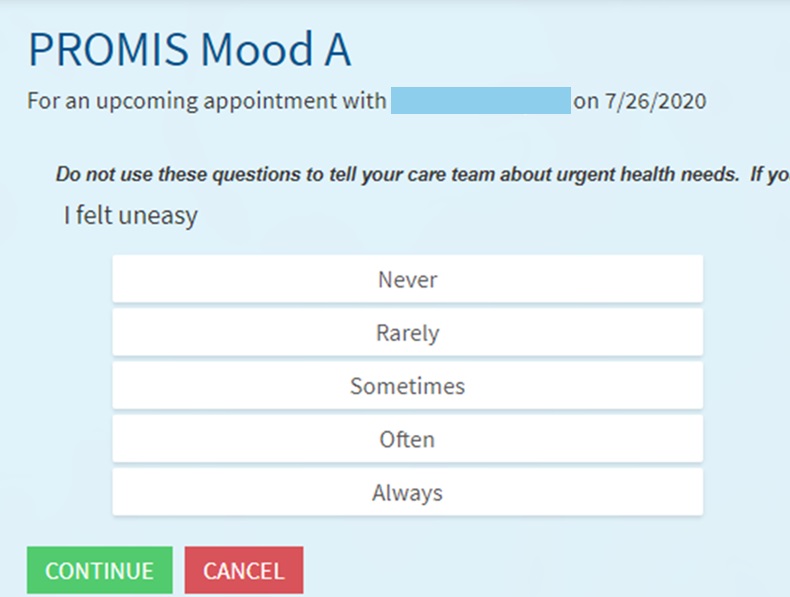

Supplement: Supplementary file 2 — Figure S1 [file CAM4-12-8860-s002.jpg]

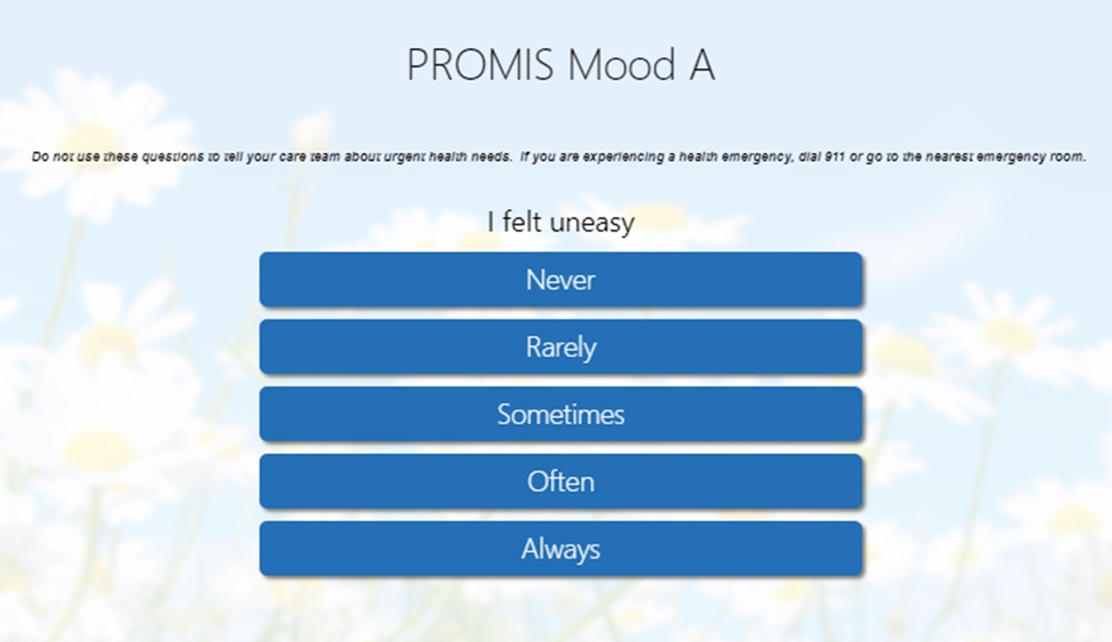

Supplement: Supplementary file 3 — Figure S2 [file CAM4-12-8860-s003.jpg]
